# Effect of Acidic Environment on the Push-Out Bond Strength of Calcium-Enriched Mixture Cement

**Published:** 2014-10-07

**Authors:** Fereshte Sobhnamayan, Safoora Sahebi, Misagh Naderi, Nooshin Sadat Shojaee, Najmeh Shanbezadeh

**Affiliations:** a *Department of Endodontics, Dental School, Shiraz University of Medical Sciences, Shiraz, Iran;*; b *Students’ Research Committee, Dental School, International Branch, Shiraz University of Medical Sciences, Shiraz, Iran;*; c *Department of Maxillofacial Medicine, Dental School, Tehran University of Medical Science, Tehran, Iran*

**Keywords:** Acidic Environment, Calcium-Enriched Mixture, CEM cement, Push-Out Bond Strength, Root-End filling

## Abstract

**Introduction:** This laboratory study was performed to evaluate the effect of different acidic pH values on the push-out bond strength of calcium-enriched mixture (CEM) cement. **Methods and Materials:** Forty-eight root dentin slices were obtained from freshly extracted single rooted human teeth and their lumen were instrumented to achieve a diameter of 1.3 mm. Then, CEM cement was mixed according to manufacturers’ instruction and placed in the lumens with minimal pressure. The specimens were randomly divided into four groups (*n*=12) which were wrapped in pieces of gauze soaked in either synthetic tissue fluid (STF) (pH=7.4) or butyric acid which was buffered at pH values of 4.4, 5.4 and 6.4. They were then incubated for 4 days at 37°C. The push-out test was performed by means of the universal testing machine. Specimens were then examined under a digital light microscope at 20× magnification to determine the nature of the bond failure. The data were analyzed using the Kruskal-Wallis test followed by Dunn’s test for pairwise comparisons. **Results: **The highest push-out bond strength (10.19±4.39) was seen in the pH level of 6.4, which was significantly different from the other groups (*P*<0.05). The values decreased to 2.42±2.25 MPa after exposure to pH value of 4.4. **Conclusion: **Lower pH value of highly acidic environments (pH=4.4), adversely affects the force needed for displacement of CEM cement; while in higher pH values (pH=6.4) the bond-strength was not affected. CEM cement is recommended in clinical situations where exposure to acidic environment is unavoidable.

## Introduction

 Root-end filling materials should seal the contents of root canal system to prevent the egress of microorganisms or their byproducts into periradicular tissues [1]. Choosing an appropriate retrograde filling material is an important factor for a successful apical surgery as well as repairing the accidental root perforations [2-5]. The ideal repair material should provide an adequate seal, be compatible with periradicular tissues, possess the ability to induce osteogenesis and cementogenesis [6], be nontoxic and adapt to the root canal walls as closely as possible [7, 8]. In addition, it is also expected to be cost-effective and easy to manipulate [1, 9]. Calcium-enriched mixture (CEM) cement is an endodontic cement consisting of different calcium compositions (i.e. calcium oxide, calcium phosphate, calcium carbonate, calcium silicate, calcium sulphate, calcium hydroxide and calcium chloride) with variable clinical applications such as root-end filling, perforation repair, vital pulp therapy and also apexification in necrotic immature teeth [10-12]. CEM Cement has high concentration of water-soluble calcium and phosphate, and immediately forms hydroxyapatite during and after setting. This cement sets in aqueous environment and is biocompatible, antibacterial and capable to form an effective seal against re-entrance of microorganisms and easy to handle. It is also able to stimulate hard tissue healing [13-15]. CEM cement shows good handling characteristics, acceptable setting time (<1 h), and has less film thickness and more flow than mineral trioxide aggregate (MTA) [10]. It also forms an effective seal [12] and is able to produce hydroxyapatite [16]. CEM cement also has antibacterial and antifungal effects against Enterococcus Faecalis and Candida Albicans similar to MTA [17, 18].

**Figure 1:**
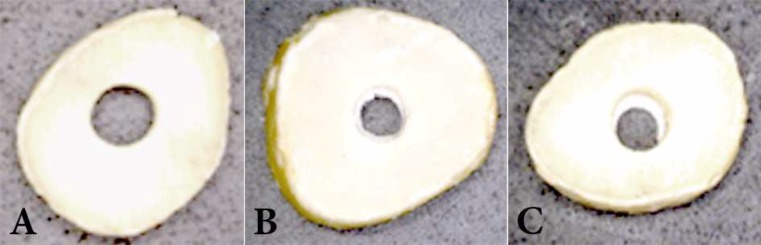
Various failure modes; A) Adhesive failure; note the clean canal wall, B) Cohesive failure within CEM, C) Mixed failure; note the CEM residual inside the canal

Reparative materials that are used as apical barriers in immature necrotic teeth or in periradicular surgery may be in contact with inflamed tissues. The pH of the abscess is as low as 5.0 [19]. This acidic environment could impede setting reaction, increase the solubility of dental materials and affect their adhesion [20, 21]. It may also impede the physical and chemical properties of MTA such as setting time [22], strength [20], hardness and sealing ability [23, 24]. Shokouhinejad *et al*. [25] proved the adverse effect of acidic environment on the push-out bond strength of MTA.

An ideal root-end filling material should adhere to dentinal walls in order to stand functional pressures on the root ends [26, 27]. Periapical pressure applied to these materials is notably high during chewing as the masticatory cycle increases the leakage of restorative materials like amalgam [26].

CEM cement as a root-end filling material may also be placed in an acidic environment in periradicular surgeries or root perforations. This laboratory study was designed to evaluate the push-out bond strength of CEM cement in different acidic environments.

## Methods and Materials

Forty-eight freshly extracted human teeth including mandibular single-rooted premolars and maxillary anterior incisors with intact crowns or minimal carious lesions were selected and stored in 0.5% chloramine-T solution at 4°C for up to 1 month before use. The teeth were horizontally sectioned in midroot area into 1.0±0.2 mm-thick slices. A diamond saw microtome (SP1600 microtome; Leica, Nußloch, Germany) was used to obtain 48 root dentin slices. The lumen of the root dentin disks were instrumented with sizes 2 to 5 Gates Glidden drills (Dentsply Maillefer, Ballaigues, Switzerland) to achieve a standardized diameter of 1.3 mm. CEM cement (BioniqueDent, Tehran, Iran) was mixed according to the manufacturer's instructions. The mixture was then placed incrementally with light pressure into the lumens of the slices. The specimens were then divided randomly into four groups (*n*=12). In group A, the slices were wrapped in pieces of gauze soaked in synthetic tissue fluid (STF) (pH=7.4). In groups B, C, and D, the specimens were wrapped in pieces of gauze soaked in butyric acid (BA) buffered at pH values of 6.4, 5.4 or 4.4, respectively. The specimens were then incubated for 4 days at 37°C.

**Table 1:** Mean (SD) of the push-out bond strength in different pH values

**pH value**	**Bond-strength [Mean (SD)]**
**4.4**	1.67 [2.42 (2.25)]
**5.4**	3.45 [4.19 (1.97)]
**6.4**	9.56 [10.19 (4.39)]
**7.4**	4.98 [5.64 (2.43)]

The push-out bond strength values were measured using a universal testing machine (Z050; Zwick/Roell Group, Ulm, Germany). The samples were placed on a metal slab with a central hole to allow the free motion of the plunger. The compressive load was applied by exerting a downward pressure on the surface of the CEM cement using a 1.00-mm diameter cylindrical stainless steel plunger at a speed of 1 mm/min. The plunger had a clearance of approximately 0.2 mm from the margin of the dentinal wall to ensure contact with the CEM bulk only. The maximum load applied on CEM cement at the time of dislodgement was recorded in Newton (N). In order to express the bond strength in MPa, the recorded value was divided by the adhesion area of root canal filling calculated according to the following formula: 2*πr*×*h*, where *r* is the root canal radius and *h* is the thickness of the dentin slice in mm. The slices were then examined under a light microscope at 20× magnification to determine the nature of the bond failure. Each sample was categorized into one of the three failure modes: adhesive failure at the CEM cement and dentin interface, cohesive failure within the CEM cement or mixed failure (Figure 1). The data were analyzed using the Kruskal-Wallis H test to compare the groups followed by Dunn’s test for pairwise comparisons.

## Results

**Figure 2:**
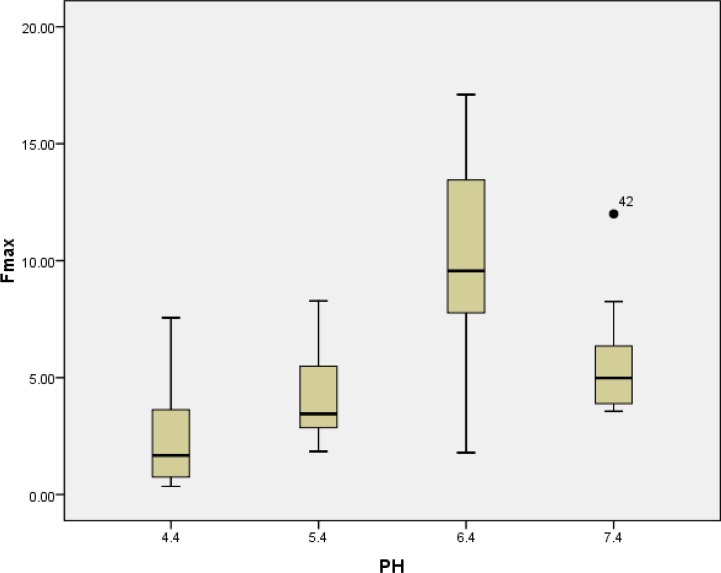
The effect of pH on the bond strength between CEM and dentin

The mean push-out bond strength after exposure to pH values of 4.4, 5.4, 6.4, and 7.4 are presented in Table 1. The greatest amount of push-out bond strength (10.19±4.39) was seen after exposure to pH of 6.4. The values decreased to 2.42±2.25 MPa after exposure to pH of 4.4. Tukey’s post hoc test revealed significant differences between group B (BA; pH=6.4) compared to other groups (*P*˂0.05). There was also a significant difference between the mean bond strength of group D (BA; pH=4.4) and group A (STF; pH=7.4) (*P*=0.04). There was not a significant difference between the mean bond strength of CEM cement in pH values of 4.4 and 5.4 (*P*=0.46). No significant difference was seen for the mean bond strength of pH 5.4 and 7.4, either (*P*=0.61) (Figure 2). The bond failure of the different groups is shown in Table 2.

**Table 2:** Type of bond failure in different pH values

**pH value**	**Failure type (%)**		
	**Adhesive**	**Cohesive**	**Mixed**
**4.4**	58.3	0	41.7
**5.4**	0	25	75
**6.4**	8.3	50	41.7
**7.4**	0	50	50

## Discussion

This laboratory study evaluated the push-out bond strength of CEM cement in different pH values and showed the highest bond strength in pH of 6.4 followed by pH values of 7.4, 5.4 and 4.4, in descending order.

An ideal root-end filling material should adhere to dentinal walls, remain unaffected by the presence of low pH levels and moisture, and tolerate dislocating forces like mechanical stresses caused by operative procedures or masticatory forces [1, 28-31]. Various methods exist to evaluate the adhesion of dental materials to dentinal wall including tensile, shear, and push-out bond strength tests [32]. Among these various methods, push-out bond test has been shown to be reliable [33].

In certain clinical situations, CEM cement may be applied in the presence of infection or inflammation. In this condition the surface of the material would be exposed to an acidic environment [19]. The application of CEM cement in lower pH might influence its physical and chemical properties. In our study, in order to stimulate the clinical conditions attributed to acidic environment and inflammation, BA was used as it has been reported to be one of the metabolic byproducts of anaerobic bacteria [34].

A few studies have been conducted on the bond strength of CEM cement. In a study on the push-out bond strength of MTA and CEM cement as root-end filling materials in root-end cavities prepared by ultrasonic technique or Er, Cr: YSGG laser, Shokouhinejad *et al.* [35] found that both materials showed significantly higher bond strength in root-end cavities that were prepared using ultrasonic technique. The bond strengths of MTA and CEM were not significantly different. In conclusion, bond strengths of MTA and CEM were comparable and higher in ultrasonically prepared cavities. 

The results of the present study showed that the mean push-out bond strength of CEM cement significantly decreased in the pH values of 4.4 and 5.4 compared to pH level of 6.4. This is partly in agreement with some other studies about MTA, like the one by Watts *et al.* [36] who reported that mixing white and gray MTA with anesthetic solution exposed to pH value of 5 significantly decreased the compressive strength of these materials. No significant difference was found in compressive strength of these two materials when mixed with sterile water and exposed to pH value of 5 or 7. Namazikhah *et al.* [23] found the greatest and the lowest surface hardness of MTA in pH value of 7.4 and 4.4, respectively. Scanning electron microscopy (SEM) showed the development of porous surface and lack of needle-like crystals in acidic environment. These porous surfaces might be formed in CEM cement in lower pH, which can be a subject of future research. In the presence of acidic environment the formation of hydroxyapatite crystals and thus the formation of hybrid layer between dentinal walls and CEM cement are likely to be impeded.

In the present study there was not a significant difference between the pH values of 5.4 and 7.4. This finding could be attributed to the small particle size of CEM cement. The greatest distribution of CEM particle sizes in one study was within 0.5-2.5 µm range which can allow penetration of particles into dentin tubules, and provide a better seal [37]. White MTA with nano particle size also showed better physical and chemical properties as a result of more resistance to acidic environment, increased surface area and less porosity [38]. The shorter setting time of CEM cement causes this material to have shorter early setting time; which is the most important period for structure formation and ion release [39]. This earlier structure formation and more ion release may also cause CEM cement to be less affected by acidic environment, although it seems that in pH values lower than 5.4 these theoretical mechanisms do not work.

In the present study, the bond failure observed in specimens exposed to pH value of 4.4 was predominantly adhesive. This kind of failure could be the result of the short storage time before bond strength evaluation. Besides the effect of time on the bond failure, very low acidic environment may also impede the formation of hybrid layer between CEM cement and dentine and cause this kind of failure. In other groups, the bond failures were mostly cohesive or mixed type which is in accordance with one study that showed bond failure of both CEM cement and MTA were predominantly of the mixed type [40], and another study that revealed the failure mode of MTA and CEM cement were predominantly of cohesive type [41]. This shows that in acidic environment, not as low as 4.4, CEM cement shows good results and is not affected.

## Conclusion

Highly acidic environment could impede the bond strength of CEM but in the pH values more than 5.4 this effect is modulated. As the pH value of abscess environment is about 5, this cement could be recommended for use in inflamed/infected tissues.
